# Does Religion Hinder Creativity? A National Level Study on the Roles of Religiosity and Different Denominations

**DOI:** 10.3389/fpsyg.2018.01912

**Published:** 2018-10-08

**Authors:** Zhen Liu, Qingke Guo, Peng Sun, Zhao Wang, Rui Wu

**Affiliations:** ^1^School of Psychology, Shandong Normal University, Jinan, China; ^2^College of Humanities and Social Science, Dalian Medical University, Dalian, China

**Keywords:** national creativity, religion, denominational differences, GDP per capital, national level

## Abstract

Creativity plays an irreplaceable role in economic and technological development. It seems that religion has a negative association with creativity. If it is true, how can we interpret the rapid development of human society with religious believers comprising 81% of global population? Based on the datasets of the World Values Survey and the Global Creativity Index, this study examined the effects of different religions/denominations on national creativity, and the moderation effect of gross domestic product per capita (GDPpc) in 87 countries. The results showed that: (1) religiosity was negatively associated with creativity at national level; (2) Proportions of Protestant and Catholic adherents in a country were both positively associated with national creativity, while proportion of Islam adherents was negatively associated with national creativity; (3) GDPpc moderated the relationships of creativity with overall religiosity, proportion of Protestant adherents, and proportion of Catholic adherents. In countries with high GDPpc, national religiosity and proportion of Islam could negatively predict national creativity, and proportion of Protestants could positively predict national creativity; in countries with low GDPpc, these relationships became insignificant. These findings suggest that national religiosity hinders creativity to a certain extent. However, some denominations (i.e., Protestant and Catholic) may exert positive influences on creativity due to their religious traditions and values. The religion–creativity relationship at national level only emerges in affluent countries.

## Introduction

Creativity, one of the unique human abilities, has been playing an irreplaceable role in human society. Technological advancement and economic growth both can benefit from creativity. For countries, creativity has been considered as a key indicator of national competitiveness (Florida, 2002). Recently, increasing literature on creativity has been conducted from a cultural perspective (Glăveanu, 2010), mostly at national level (e.g., Rinne et al., 2013; Efrat, 2014; Kaasa, 2016). Religion has strong power in shaping societal and individual outcomes (Herbig and Dunphy, 1998; Chan-Serafin et al., 2013; Okulicz-Kozaryn, 2015), but few studies have examined the effect of religion on national creativity. Some researchers posit that religion hinders creativity, because rules and traditions are over stressed by religion while creativity requires people to challenge traditions and rules to seek a breakthrough (Gino and Wiltermuth, 2014; Okulicz-Kozaryn, 2015). But some other researchers argue that religion may be beneficial for creativity because religion cultivates personal virtues (e.g., hard-working) and cognitive schema that are positively associated with creativity (Day, 2005; Assouad and Parboteeah, 2018). In fact, empirical studies tend to support a negative association between religion and creativity (e.g., Dollinger, 2007; Bénabou et al., 2013, 2015; Okulicz-Kozaryn, 2015). However, if religion hinders creativity, it seems paradoxical that rapid economic and technological development can occur in this world where more than 81% of the population is religious (according to the 6th waves of World Value Survey, WVS).

Previous literature suggests that different religions/denominations have dissimilar effects on prosocial behaviors (Prouteau and Sardinha, 2015), trust (Dingemans and Ingen, 2015), and entrepreneurship (Dana, 2009). National economic situation has been found to have a moderating effect on the relationship between religion and outcomes, such as values (Saroglou et al., 2004) and prosociality (Guo et al., 2018). We speculated that the different findings on religion–creativity relationship and the paradox between rapid development and numerous religious populations can be accounted for by the dissimilar effects of religious denominations, as well as the moderation effect of economy. Thus, using datasets of WVS and the Global Creativity Index (GCI), we conducted this national-level study to explore the influences of different denominations on creativity, and the moderating role of economic factor in the religion–creativity relationship.

### Creativity at National Level

Glăveanu (2010) classified the research on creativity into three paradigms, namely, He-paradigm, I-paradigm, and We-paradigms. He-paradigm is the earliest strategy for the exploration of creativity, which focuses on outstanding ability and fertility of the genius. This paradigm always considers creativity exclusive to the genius group. With the increase of creativity studies, I-paradigm emerges, which starts to investigate creativity of ordinary individuals rather than genius. In this stage, the relationship between creativity and personal attributes (such as personality and cognition) attracts the interest of many researchers. In the last few decades, We-paradigm, which focuses on social psychology of creativity, has become prevalent. Researchers adopting this paradigm, have gradually realized the great influence of sociocultural factors on creativity (Glăveanu, 2010), and tend to understand creativity in particular contexts (e.g., Rinne et al., 2013; Kaasa, 2016).

A growing body of literature has examined the relationship between culture variables, especially the Hofstede (1980) cultural values, and national creativity. Using data of 33 countries, Shane (1993) explored the relationship between Hofstede’s cultural values and per capita numbers of trademarks which was used as indicator of national creativity. He found that national rate of innovation was negatively associated with power distance and uncertainty avoidance, but was positively connected with individualism. These findings were completely replicated by Efrat (2014) who used more comprehensive indicators of national creativity, including patents, scientific and technical journal articles, and high-technology exports. However, using a dataset for 43 countries, Rinne et al. (2013) found that only individualism was positively related to indicators of national creativity, the GCI and the Design and Creativity Index. The negative effects of power distance and uncertainty avoidance on national creativity had not been replicated.

### Religion and Creativity

There have always been controversies over the religion–creativity relationship. Existing literature supports two opposite standpoints: religion hinders creativity and religion facilitates creativity, which were, respectively, called “hinder hypothesis” and “facilitate hypothesis” in this study.

Okulicz-Kozaryn (2015) argued that religion requires people to follow traditions and discourages people to embrace diversity. Thus most of religious followers tend to be conservative individuals who are more likely to be less creative (Dollinger, 2007). Moreover, creativity is associated with challenging traditions and rules, and tolerance of diversity, which are discouraged by most religious traditions. Brenkert (2009) pointed that rule breaking was a feature of creativity and innovation. Gino and Wiltermuth (2014) found that dishonest individuals tend to be more creative. They further proposed that dishonesty and creativity both involved rule-breaking. These findings may indirectly support the “hinder hypothesis” because religious people are usually more honest and are more likely to follow the rules than their secular counterparts (Saroglou, 2010; Okulicz-Kozaryn, 2015). Meanwhile, religiosity has been found to be positively related with conformity that is disruptive to creativity, but negatively with self-direction that is conductive to creativity (Schwartz and Huismans, 1995). Given the above, it seems reasonable to assume that religion hinders creativity.

However, Assouad and Parboteeah (2018) held that the believers’ particular traits (e.g., self-control, honest, spirit of cooperation, and hard-working) fostered by religions can contribute to creativity. By cultivating these virtues in the adherents, religion can build a positive environment and network for creativity and entrepreneurship (Dana, 2009; Assouad and Parboteeah, 2018). Day (2005) further proposed that religion can facilitate creativity through different mechanisms. First, people in religious activities can learn to view their experiences in a new way. Second, religious faith can enrich followers’ schemas which provide more ways for organizing information. Third, religious activities can facilitate internal loci of control, which is associated with more effective problem solving (Day, 2005). Recently Shen et al. (2017) have found a positive relationship between morality and creativity, providing indirect evidence for the “facilitate hypothesis.” That is, religion cultivates morality, and morality is positively associated with creativity.

To date there have been only a few researches studying the religion–creativity association at aggregate level. Okulicz-Kozaryn (2015) investigated the relationship between religiosity and creativity at local level (i.e., across United States counties). They found that local religiosity (indicated by adherence per population and church density) correlated significantly and negatively with local creativity (indicated by creative class and patent number), supporting the “hinder hypothesis.” Bénabou et al. (2013) also found negative relationships between religion and creativity both across US states and across the world. Using dataset for 30 countries, Assouad and Parboteeah (2018) found that the normative aspect of religion had a positive relation with firm-level innovation, which supports the “facilitate hypothesis.” But they also found that the regulatory aspect of religion was negatively related to firm-level innovation, while the normative aspect of religion showed no relationship with creativity.

Contrary to the scarcity of direct research, there are a greater number of indirect studies on the religion–creativity association, mostly supporting the “hinder hypothesis.” Scientists are considered to be the most creative people both by the public and by the academia. Scientists tend to have lower level of religiosity and smaller proportion of adherents than the general population (Larson and Witham, 1998; Ecklund and Scheitle, 2007). Furthermore, the proportion of believers among scientists has been observed to be on a downward trend (Larson and Witham, 1998). Besides, the I-paradigm also provides indirect evidence for the “hinder hypothesis.” For example, Dollinger (2007) found that highly conservative individuals, who had poor performances on creativity, tended to use religiosity as the common theme in a photo essay task. Using the data of WVS, Bénabou et al. (2015) found that individual religiosity negatively predicted pro-innovation attitudes and positively predicted anti-innovation attitudes even when numerous socio-demographic variables were controlled.

### The Present Study

Culture is the set of customs, traditions, and values shared by people in a society or a community (Kaasa, 2016). Herbig and Dunphy (1998, p. 18) defined religion as “a socially shared set of beliefs, ideas, and actions which……is believed to affect the course of natural and human events.” From these definitions it is easy to find that religion and culture are similar constructs. Religion and culture shape each other, and are part of each other (Ronen and Shenkar, 2013). Culture’s influence on creativity has been revealed by numerous studies (e.g., Shane, 1993; Efrat, 2014), while the relationship between religion and creativity/innovation has not been soundly addressed. This is the reason why this study was designed and conducted.

Using datasets of GCI and WVS, we explored the relationship between religion and national creativity. GCI covers three aspects of national creativity, namely technology, talent, and tolerance (Florida, 2002, 2014). Thus it is an indicator of national creativity more comprehensive than that used by Bénabou et al. (2013), as well as that used by Assouad and Parboteeah (2018). Considering the results of direct and indirect research on religion and creativity, we propose that religiosity is negatively related with creativity at national level (*Hypothesis 1*).

Religion plays an essential role in influencing individual even social/national outcomes. And creativity is a vital factor in shaping social development and economic growth of each country, whether it is religious or not (Raghupathi and Raghupathi, 2017). However, existing literature seemingly shows that religion is not beneficial for creativity, despite there are still different voices. Religious population comprises more than 81% of the world’s population. And the overwhelming majority of countries in the world are religious. If the conclusion is in accordance with reality, the technological development and economic growth in our world should have not been so rapid. What causes this paradoxical phenomenon? We propose that different effects of religions/denominations and moderation effect of economy may be solutions to this perplexing question.

Berry (1999) investigated the general relationship between religious backgrounds and creativity, using about 1,400 outstanding achievers in art- or science-related areas. He found that the achievers in science areas were mostly from Protestant background, while the achievers in art-related areas were mostly from Catholic background. Dana (2009) also found that religion may have both positive and negative impact on entrepreneurship, which may differ across religious denominations. Various religions value entrepreneurship differently, and contribute to different networks (including credit, employment, information, and supply networks of co-religionists) that affect entrepreneurship (Dana, 2009). These findings show that the effects of religions on national creativity may depend on teachings and values of different religions/denominations. Previous literature suggests that the proportion of adherents in a population can be considered as an indicator of the religious culture (e.g., Okulicz-Kozaryn, 2015; Einolf, 2017). And the tradition and values maintained and promoted by a religion/denomination could pervade religious boundaries and exert influences on the whole society (Lam, 2006). According to samples and variables in the dataset of WVS, this study used five religious denominations (i.e., Protestant, Orthodoxy, Catholic, Islam, and Buddhist). We propose that various religions/denominations have different relationships with national creativity.

According to the Protestant work ethic (Weber, 1930) valuing hard work, discipline, and frugality (Inglehart and Oyserman, 2004), and the finding that a greater number of achievers in science-related areas come from Protestant background (Berry, 1999), we hypothesize that Protestant culture is positively related with national creativity (*Hypothesis 2a*). Due to the fact that Catholic also has a tradition encouraging hard work and thrift which can translate into economic success (Andersen et al., 2017), and the finding that achievers from Catholic background have more creativity in arts than counterparts from Protestant background (Berry, 1999), we hypothesize that Catholic culture may also have a positive relation with national creativity (*Hypothesis 2b*). What effect Orthodoxy has on creativity is not hypothesized in this study because the Orthodoxy–creativity relationship has seldom been studied by existing literature. With respect to Muslim, we hypothesize its relationship with national creativity is negative (*Hypothesis 2c*), because determinism is deeply embedded in Islam culture (Herbig and Dunphy, 1998; Westwood and Low, 2003). Furthermore, traditional interpretations of Islam are not compatible with the development of science, which also hinders creativity. It is a little difficult to deduce the Buddhism–creativity relationship. On one hand, Buddhism may “de-emphasize materialism and encourage acceptance and quietude” (Westwood and Low, 2003, p. 242), suggesting that Buddhism does not encourage change and innovation. On the other hand, Buddhism emphasizes impermanence and recommends its adherents to engage in mindfulness and meditation practice, which can improve creativity (Colzato et al., 2012; Ding et al., 2014; Berkovich-Ohana et al., 2017). These effects of Buddhism may operate in the opposite directions, leading us to hypothesize that there is no relationship between Buddhism culture and national creativity (*Hypothesis 2d*).

Creativity and economy influence one another (Rinne et al., 2013; Raghupathi and Raghupathi, 2017). It seems that economic factors should be taken into consideration when the religion–creativity relation is examined. Previous literature has indicated a moderating effect of economy in the religiosity–prosociality relationship at national level (Guo et al., 2018). This suggests that roles religion plays may vary according to different levels of economic development across countries (Saroglou et al., 2004). Therefore gross domestic product per capita (GDPpc) was introduced as a moderator in this study to explore the detailed relationship between religion and creativity.

The relationship between economy and creativity/innovation is bidirectional (Rinne et al., 2013; Raghupathi and Raghupathi, 2017). However, in the initial phase of economic development in a country, economy can be developed prior to or even be independent with innovation. In developing countries, the patents of foreigners take up a considerable proportion (Raghupathi and Raghupathi, 2017), and technology mostly relies on “spillovers” of developed countries (Fagerberg et al., 2010). In this case, the religion–creativity relationship may be too weak to be observed. But with full development of economy, the impact of religion on creativity/innovation should become evident. This is because that in developed countries creativity/innovation is an essential factor for economic development (Fagerberg et al., 2010). In this case, intrinsic relationship of religion with national creativity will rise to the surface. Thus, we proposed another hypothesis that the association of religions/denominations and national creativity may be moderated by GDPpc. Specifically, the religion–creativity relationship would be weaker in low (even disappear) relative to high GDPpc countries (*Hypothesis* 3).

## Materials and Methods

A nation-level design was applied in this study, regarding a country as a unit of analysis (e.g., Rinne et al., 2013; Kaasa, 2016; Guo et al., 2018). Finally there were 87 countries in our analyses with data available for all research variables.

### Independent Variables

Religious variables were provided by the WVS^1^ (Inglehart et al., 2014), a major cross cultural survey on beliefs and values. Since 1981, WVS has been conducted for six waves, generating a dataset including about 100 countries. Datasets for six waves were all involved in this study.

According to previous research (Bénabou et al., 2013; Guo et al., 2018), overall religiosity of a country/region was measured with four items of WVS: church attendance, importance of deity, importance of religion, and religious faith for children. The item, “Apart from weddings and funerals, how often do you attend religious services these days?” with an 8-point scale ranging from “several times a day” to “never,” was used to capture the church attendance. The importance of deity was measured by the question “How important is deity in your life” using a 10-point scale (1 = “not at all important,” 10 = “very important”), where the particular deity depends on the participants’ religion. The importance of religion was measured by the item, “How important is religion in your life” with a 4-point scale ranging from “very important” to “not at all important.” After reverse-scoring the negatively worded items, each score of these items was averaged according to country/region (individual-level Cronbach’s α = 0.70). The religious faith for children was measured by the proportion of participants in each country who chose religious faith as one of the important qualities (up to five) for children. Then the four scores were standardized at country level and combined into the indicator of overall religiosity of each country (national-level Cronbach’s α = 0.82). It should be noted that both religious and irreligious respondents answered these four items. We computed the overall religiosity indicator for all valid respondents rather than for only adherents of the five denominations.

Religious denominations in WVS have been classified in great detail. Following previous literature (Berger, 2006), we identified five religions/denominations (Protestant, Orthodoxy, Catholicism, Muslim, and Buddhism) and merged their sub-denominations (see **Table 1**). The number of believers of each denomination was calculated according to answers to the item “Do you belong to a religion or religious denomination? If yes, which one.” Then the numbers for believers of the five denominations were divided by the valid sample sizes, respectively, to indicate the percentages of five denominations in each country.

**Table 1:** Religious denominations and sub-denominations.

Denomination	Sub-denomination
Protestant	Anglican; Baptist; Christian Reform; Evangelical; Methodists; Pentecostal; Presbyterian; Protestant; Seven Day Adventist; The Church of Sweden; Dutch Reformed; Reformed Churches in the Netherlands; Evangelical/Apostolic Faith Mission
Catholicism	Catholic: Does not follow rules; Greek Catholic; Roman Catholic
Orthodoxy	Orthodox
Muslim	Al-Hadis; Muslim; Shia; Sunni
Buddhism	Buddhism

### Dependent Variable

National creativity was taken from the research of Florida et al. (2015). In their study on GCI, an indicator of nation-level creativity, of 139 countries across the world, was measured on a 3Ts (Technology, Talent, and Tolerance) model of creativity (Florida, 2002, 2014). GCI is a broad-based measure of national creativity that includes research and development investment, patent applications, creative class, educational attainment, and attitudes toward minorities. Thus, compared to indicators used in previous research, such as patents per capita (e.g., Bénabou et al., 2013) and proportion of individuals in creative occupations (e.g., Okulicz-Kozaryn, 2015), GCI can be considered as a more comprehensive measure of national creativity.

### Moderator Variable

GDPpc, which is usually used as an economic variable in national-level studies, was taken from the World Bank Open Data^2^. The last WVS wave was conducted from 2010 to 2014, so the indicator of GDPpc was calculated by averaging the data collected during 2010–2014. A logarithm transformation was applied to GDPpc in order to yield normally distributed data.

### Control Variables

Intelligence is significantly associated with creativity (for a review, see Sternberg and O’Hara, 1999). Cinnirella and Streb (2017) argued that religious tolerance, measured by the religious pluralism index (RPI), had a positive effect on creativity and innovation. Therefore, national IQ and RPI were used as controls in this study. National IQ was obtained from a research focusing on intelligence and human capital (Meisenberg and Lynn, 2011). In this research, the missing data of human capital were extrapolated by national IQ, as a high correlation between them (*r* = 0.981). Thus, the missing data of IQ were substituted by the Human Capital reported by Meisenberg and Lynn (2011) in this study. Scores for national RPI were calculated using the formula 1-Σ^*N*^_i_ = _1_$\pi_{\text{i}}^{2}$ (Cinnirella and Streb, 2017), where π_i_ refers to the percentage of individuals who believe Protestant, Orthodoxy, Catholicism, Muslim, Buddhism, or other religions in each country, respectively.

## Results

Descriptive statistics for 87 countries/regions, including the numbers of valid participants and believers of different denominations, scores for overall religiosity, national IQ, GDPpc, and RPI were presented in **Table 2**.

**Table 2:** Descriptive statistics at national level (*N* = 87).

country	*N*	Catholicism	Protestant	Orthodoxy	Muslim	Buddhism	Religiosity	GCI	IQ	RPI
Albania	1994	650	184	204	706	6	−1.02	0.20	82.90	0.75
Algeria	2482	0	0	0	2476	0	3.77	0.28	82.80	0.00
Argentina	6371	4872	131	29	4	76	−0.39	0.68	96.00	0.41
Armenia	3056	14	6	11	1	2	−1.20	0.27	92.00	0.23
Australia	4858	1209	1727	83	32	63	−4.17	0.97	98.00	0.81
Azerbaijan	2991	2	5	53	2794	0	−0.81	0.24	84.80	0.13
Bangladesh	3021	17	2	1	2684	10	5.06	0.32	81.00	0.20
Belarus	3552	322	32	2264	6	0	−3.24	0.60	95.10	0.59
Bosnia	1185	154	1	248	485	0	−0.45	0.25	94.00	0.77
Brazil	4582	2916	775	117	3	11	2.31	0.67	87.00	0.56
Bulgaria	2048	14	9	1285	238	2	−3.85	0.51	92.50	0.59
Burkina Faso	1517	473	120	3	818	0	4.54	0.38	71.00	0.60
Canada	4030	1530	729	39	59	24	−1.49	0.92	100.00	0.81
Chile	5647	3624	531	142	0	1	0.39	0.61	91.00	0.58
China	6138	30	151	0	117	300	−8.33	0.46	105.50	1.00
Colombia	10545	8224	535	127	2	2	2.36	0.41	83.50	0.39
Croatia	1174	989	4	14	14	0	−2.11	0.48	99.00	0.29
Cyprus	2042	11	3	1415	498	0	0.93	0.45	91.50	0.46
Czechia	1993	797	75	0	0	0	−6.58	0.61	98.00	0.84
Dominican Republic	409	245	48	0	0	0	2.58	0.92	82.00	0.63
Ecuador	1201	753	142	0	0	0	2.70	0.53	88.00	0.59
Egypt	6050	0	0	0	5687	0	4.87	0.20	81.00	0.11
El Salvador	1254	738	288	0	0	28	4.17	0.25	78.90	0.60
Estonia	2509	34	215	516	6	6	−5.86	0.63	99.00	0.95
Ethiopia	1482	23	291	971	158	1	3.59	0.30	68.50	0.52
Finland	2991	325	2097	30	63	0	−4.09	0.92	97.00	0.49
France	994	417	22	2	47	5	−5.59	0.82	98.00	0.82
Georgia	4698	42	4	4225	166	2	1.65	0.45	86.50	0.19
Germany	6034	1375	1941	47	148	8	−5.12	0.84	99.00	0.84
Ghana	3047	528	1748	193	404	1	5.35	0.07	70.00	0.62
Great Britain	1012	113	293	3	43	4	−3.98	0.88	100.00	0.90
Guatemala	995	559	310	0	2	1	4.60	0.45	79.00	0.59
Hong Kong	2243	67	102	0	2	273	−5.79	0.72	108.00	0.97
Hungary	3023	1827	679	17	6	0	−4.24	0.67	96.50	0.58
India	9976	167	86	40	983	119	1.00	0.29	82.00	0.30
Indonesia	3007	65	136	0	2785	0	5.79	0.20	87.00	0.14
Iran	5187	0	0	0	5081	0	3.71	0.48	83.50	0.04
Iraq	6207	16	2	9	6159	0	3.98	0.03	87.00	0.02
Italy	1011	885	0	0	0	2	0.34	0.72	97.00	0.23
Japan	7367	45	55	106	0	2924	−5.14	0.71	105.00	0.84
Jordan	3622	33	8	23	3499	0	4.52	0.38	84.00	0.07
Kazakhstan	1502	15	10	400	756	2	−2.88	0.36	84.70	0.68
Kyrgyzstan	2534	9	17	170	2111	3	−0.60	0.24	74.40	0.30
Latvia	1127	222	233	217	4	1	−4.36	0.56	96.10	0.88
Lebanon	1129	261	13	133	622	0	1.30	0.32	82.00	0.62
Lithuania	977	778	20	42	1	2	−2.21	0.49	92.00	0.36
Macedonia	2032	10	5	1084	505	0	−0.89	0.39	90.50	0.65
Malaysia	2498	84	55	0	1509	461	3.79	0.46	88.50	0.58
Mali	1503	27	8	1	1426	1	4.34	0.35	69.50	0.10
Mexico	10729	7957	779	39	5	8	1.78	0.41	88.00	0.44
Moldova	2950	40	49	2660	2	0	−0.37	0.26	92.50	0.19
Montenegro	317	25	0	208	68	0	−3.53	0.52	85.80	0.51
Morocco	3650	2	1	1	3635	0	5.11	0.18	84.00	0.01
Netherlands	2810	591	316	57	53	6	−5.74	0.89	100.00	0.94
New Zealand	2897	410	1470	3	12	15	−4.62	0.95	99.00	0.71
Norway	2142	25	1590	10	17	6	−5.46	0.88	100.00	0.45
Pakistan	3932	0	0	0	3320	0	5.23	0.24	84.00	0.29
Peru	5360	4178	567	0	1	3	2.11	0.42	85.00	0.38
Philippines	3593	2707	140	0	123	0	4.33	0.49	90.00	0.42
Poland	3086	2914	28	28	0	1	1.89	0.52	95.00	0.11
Romania	4462	258	243	3908	9	3	1.96	0.43	91.00	0.23
Russia	8374	18	55	4193	366	21	−4.76	0.58	96.50	0.75
Rwanda	3034	1639	753	32	305	5	2.49	0.14	76.00	0.64
Saudi Arabia	1499	0	0	0	1457	0	3.64	0.36	79.00	0.05
Singapore	3480	197	376	0	557	1055	−0.66	0.90	108.50	0.83
Slovakia	1557	1155	149	3	0	0	−1.83	0.48	98.00	0.44
Slovenia	3067	2085	49	58	41	3	−3.73	0.82	96.00	0.54
South Africa	15970	2054	7450	109	625	25	2.70	0.56	72.00	0.72
South Korea	6999	1050	1430	25	7	1710	−2.92	0.66	106.00	0.88
Spain	6256	5036	37	9	7	10	−3.56	0.81	97.00	0.35
Sweden	3192	52	2224	13	36	1	−6.55	0.92	99.00	0.51
Switzerland	3708	1612	1594	7	25	1	−2.84	0.82	101.00	0.62
Tanzania	1162	330	219	58	469	0	5.30	0.13	72.50	0.72
Thailand	2729	7	2	0	65	2639	0.56	0.37	88.00	0.06
Trinidad and Tobago	1977	400	847	6	125	5	3.72	0.43	86.70	0.72
Tunisia	1205	0	0	0	1205	0	3.82	0.26	84.00	0.00
Turkey	8259	24	13	3	7826	0	2.02	0.35	88.50	0.10
Uganda	1002	356	453	8	169	0	4.62	0.20	72.00	0.64
Ukraine	5108	358	44	3273	17	8	−2.66	0.52	95.00	0.58
United States	6018	1364	1800	25	19	31	0.84	0.95	98.00	0.82
Uruguay	2972	972	211	0	0	2	−3.78	0.69	96.00	0.89
Uzbekistan	1490	1	4	45	1426	1	−1.87	0.29	79.60	0.08
Venezuela	2366	1777	155	2	0	2	1.87	0.47	84.00	0.43
Viet Nam	2491	151	26	1	1	383	−5.22	0.38	94.00	0.76
Yemen	1000	0	0	0	1000	0	4.47	0.11	83.00	0.00
Zambia	1500	513	694	2	20	2	3.90	0.10	75.00	0.65
Zimbabwe	2498	491	1449	10	15	2	4.50	0.11	71.50	0.61

*GCI, Global Creativity Index; IQ, intelligence quotient; RPI, religious pluralism index.*

National-level correlations among variables in the present study were shown in **Table 3**. The overall religiosity was negatively related to GDPpc, GCI, IQ, and PRI. Proportion of Catholics had a positive correlation with GCI. Proportion of Protestants was positively correlated with GCI and PRI. In addition, proportion of Muslims was negatively connected with GCI, GDPpc, IQ, and RPI.

**Table 3:** Correlations among key variables at national level (*N* = 87).

	*M*	*SD*	1	2	3	4	5	6	7	8	9	10
1. Religiosity	0	3.78	1									
2. Catholicism	0.25	0.29	0.04	1								
3. Protestant	0.13	0.19	−0.08	0.02	1							
4. Orthodoxy	0.11	0.23	−0.11	−0.30^∗∗^	−0.22^∗^	1						
5. Islam	0.25	0.37	0.50^∗∗∗^	−0.48^∗∗∗^	−0.37^∗∗∗^	−0.16	1					
6. Buddhism	0.03	0.12	−0.09	−0.17	−0.11	−0.11	−0.11	1				
7. GCI	0.49	0.25	−0.65^∗∗∗^	0.22^∗^	0.28^∗∗^	−0.09	−0.53^∗∗∗^	0.06	1			
8. GDPpc	8.97	1.32	−0.63^∗∗∗^	0.17	0.20	−0.12	−0.39^∗∗∗^	0.09	0.82^∗∗∗^	1		
9. IQ	89.10	9.70	−0.77^∗∗∗^	0.08	−0.04	0.01	−0.44^∗∗∗^	0.19	0.72^∗∗∗^	0.80^∗∗∗^	1	
10. RPI	0.50	0.28	−0.55^∗∗∗^	0.09	0.35^∗∗∗^	−0.02	−0.64^∗∗∗^	0.02	0.49^∗∗∗^	0.35^∗∗∗^	0.40^∗∗∗^	1

*GCI, Global Creativity Index; GDPpc, gross domestic product per capita; IQ, intelligence quotient; RPI, religious pluralism index. ^∗^p < 0.05, ^∗∗^p < 0.01, ^∗∗∗^p < 0.001.*

Then a hierarchical regression was conducted to further explore the religion–creativity relationship, controlling IQ and RPI. IQ and RPI as the controls were entered in Step 1, and overall religiosity or denominational cultures (indicated by proportions of different religious denominations) were entered in Step 2 (see **Tables 4**, **5**). **Table 4** showed that RPI and IQ could significantly predict GCI. But religiosity no longer had a significant effect on GCI when RPI and IQ were controlled. As **Table 5** shown, only Protestant proportion and Catholic proportion could positively predict GCI, which is consist with the correlation analysis, but the negative Muslim–GCI relationship disappeared in the regression model.

**Table 4:** Regression analysis of the relationship between overall religiosity and GCI (*N* = 87).

	Model 1		Model 2	
	*b*	*t*	*b*	*t*
RPI .	0.21^∗∗^	3.04	0.18^∗^	2.40
IQ	0.02^∗∗∗^	7.91	0.01^∗∗∗^	4.88
Religiosity			−0.01	−0.86
Δ*R^2^*	0.56^∗∗∗^		0.00	

*IQ, intelligence quotient; RPI, religious pluralism index. ^∗^p < 0.05, ^∗∗^p < 0.01, ^∗∗∗^p < 0.001.*

**Table 5:** Regression analysis of the relationships between denominations and GCI (*N* = 87).

	Model 1		Model 2		Model 3	
	*b*	*t*	*b*	*t*	*b*	*t*
RPI	0.21^∗∗^	3.04	0.11	1.57	0.10	1.50
IQ	0.02^∗∗∗^	7.91	0.02^∗∗∗^	8.96	0.02^∗∗∗^	9.08
Catholicism				0.13^∗^	2.33
Protestant			0.34^∗∗∗^	3.50	0.35^∗∗∗^	3.61
Orthodoxy						
Islam						
Buddhism						
Δ*R^2^*	0.562^∗∗∗^		0.06^∗∗∗^		0.02^∗∗∗^	

*IQ, intelligence quotient; RPI, religious pluralism index. Denominational cultures were put in Step 2 using a stepwise method, with the regression coefficients of excluded variables not being presented. ^∗^p < 0.05, ^∗∗^p < 0.01, ^∗∗∗^p < 0.001.*

To test the moderating effect of GDPpc on the relationships between religion and GCI, Hayes (2013) PROCESS macro for SPSS was employed. Six moderation analyses were conducted in Model 1 with 5,000 bootstrap samples. The detailed results were presented in **Table 6**.

**Table 6:** Moderating effects of GDPpc on relationships between religion and creativity (*N* = 87).

	Independent variable → GCI				GDPpc → GCI				interaction → GCI				*R*^2^
	*b*	*SE*	CI		*b*	*SE*	CI		*b*	*SE*	CI		
Religiosity	−0.01	0.01	[−0.34, 0.83]		0.12^∗∗∗^	0.02	[0.09, 0.16]		−0.01^∗^	0.00	[−0.01, −0.00]		0.74^∗∗∗^
Catholicism	0.06	0.05	[−0.05, 0.16]		0.13^∗∗∗^	0.02	[0.09, 0.16]		0.08	0.06	[−0.04, 0.19]		0.73^∗∗∗^
Protestant	0.02	0.10	[−0.19, 0.23]		0.11^∗∗∗^	0.02	[0.07, 0.15]		0.15^∗^	0.06	[0.04, 0.26]		0.75^∗∗∗^
Orthodoxy	−0.07	0.05	[−0.16, 0.03]		0.12^∗∗∗^	0.02	[0.08, 0.16]		−0.14	0.04	[−0.22, −0.06]		0.74^∗∗∗^
Muslim	−0.23^∗∗∗^	0.05	[−0.33, −0.12]		0.12^∗∗∗^	0.02	[0.09, 0.15]		−0.17^∗∗∗^	0.04	[−0.24, −0.10]		0.80^∗∗∗^
Buddhism	−0.05	0.30	[>0.066, 0.55]		0.12^∗∗∗^	0.02	[0.09, 0.16]		−0.08	0.19	[−0.45, 0.30]		0.72^∗∗∗^

*CI, 95% confidence interval; GCI, Global Creativity Index; GDPpc, Gross Domestic Product per capita. ^∗^p < 0.05, ^∗∗^p < 0.01, ^∗∗∗^p < 0.001.*

**FIGURE 1:**
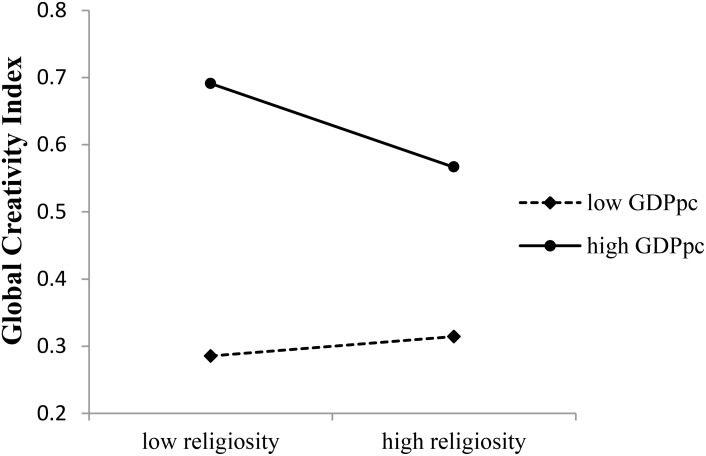
Moderating effects of GDPpc on the religiosity–creativity association.

GDPpc moderated the relationship between overall religiosity and GCI (see **Table 4**). As **Figure 1** shown, in countries with high GDPpc, the overall religiosity could negatively predict GCI (*b* = −0.016, *t*(81) = −2.023, *p* = 0.046, 95% confidence interval, CI = [−0.033, −0.000]), but in countries with low GDPpc, the predictive effect of overall religiosity on GCI was not significant (*b* = −0.004, *t*(81) = 0.522, *p* = 0.603, 95% CI = [−0.011, 0.018]).

**FIGURE 2:**
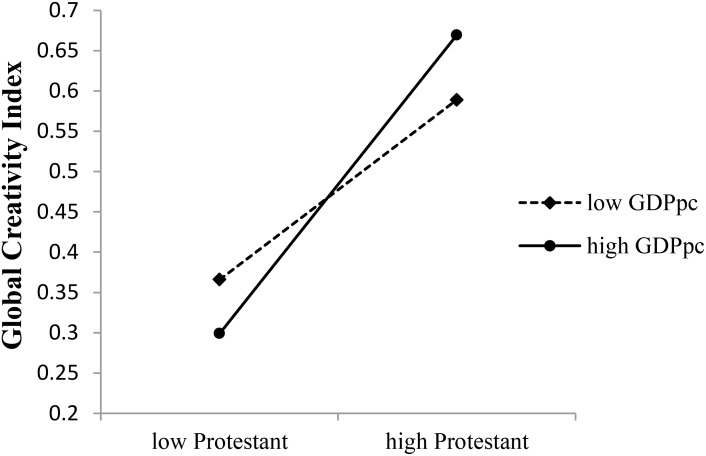
Moderating effects of GDPpc on the Protestant–creativity association.

The interaction between GDPpc and proportion of Protestants in a country positively predicted GCI (see **Table 4**). As shown in **Figure 2**, in countries with high GDPpc, proportion of Protestants could positively predict GCI (*b* = 0.214, *t*(81) = 2.302, *p* = 0.024, 95% CI = [0.029, 0.400]), while in countries with low GDPpc, the predictive effect was not significant (*b* = −0.178, *t*(81) = −1.143, *p* = 0.257, 95% CI = [−0.488, 0.132]).

**FIGURE 3:**
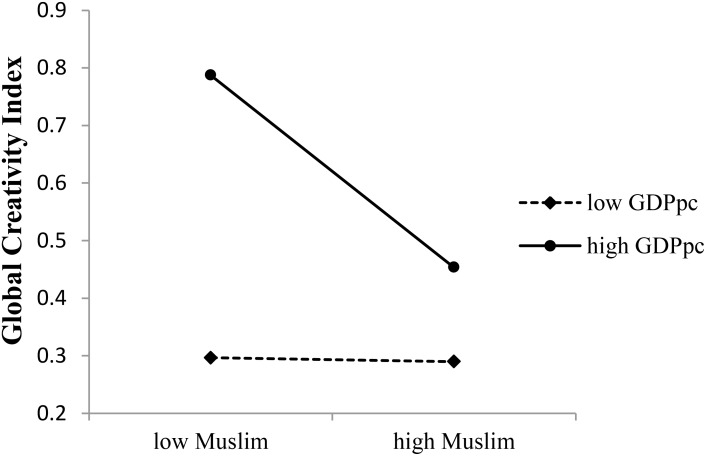
Moderating effects of GDPpc on the Muslim–creativity association.

In contrary, the interaction between GDPpc and proportion of Muslims in a country negatively predicted GCI (see **Table 4**). As shown in **Figure 3**, in countries with high GDPpc, proportion of Muslims could negatively predict GCI (*b* = −0.448, *t*(81) = −5.071, *p* < 0.001, 95% CI = [−0.624, −0.272), while in countries with low GDPpc, proportion of Muslims failed to predict GCI (*b* = −0.009, *t*(81) = −0.193, *p* = 0.847, 95% CI = [−0.104, 0.085]).

## Discussion

Creativity exerts strong influences on society, economy, science, and technology. The effect of culture on national creativity and innovation has been found by numerous studies (e.g., Efrat, 2014; Kaasa, 2016). Religion has a complex interaction with culture (Ronen and Shenkar, 2013), and is essential for human society. This study explored the effects of different religions/denominations on national creativity, and revealed some intriguing and innovative findings.

### Overall Religiosity and National Creativity

Consistent with previous findings (Bénabou et al., 2013; Okulicz-Kozaryn, 2015), correlation analysis in this study showed that overall religiosity had a negative association with GCI. Moderation analysis further indicated that this relationship was significant only in countries with high GDPpc, but disappeared in countries with low GDPpc. However, hierarchical regression analysis illustrated that religiosity failed to predict GCI when national level IQ and RPI were controlled. Therefore hypothesis 1, namely “hinder hypothesis,” was partly supported.

Religion and creativity in some sense are opposite in nature. Religion is about obedience and conformity to traditions (Schwartz and Huismans, 1995), while creativity is about challenge and change (Brenkert, 2009; Gino and Wiltermuth, 2014; Okulicz-Kozaryn, 2015). Religion is associated with conservatism (Dollinger, 2007), prescribing inheritance and protection of religious traditions. Common beliefs, values, and religious practices yield particular patterns shared by believers in a religious community (Dana, 2009). Moreover, religion emphasizes rules and traditions. Compared to secular people, it is more difficult for religious people to accept creative and innovative ideas that challenge rules and traditions. On the contrary, creativity requires a critical and doubtful spirit that traditions and existing relationships in the world are challengeable (Brenkert, 2009). Strong endorsement of obedience and conformity to traditions among religious adherents can create a conservative atmosphere in the whole society and exert a negative influence of creativity. Creativity is a social or situational phenomenon (Okulicz-Kozaryn, 2015). On one hand, creative ideas usually come from social interaction, allowing different ideas to collide and interact; on the other hand, whether one idea/activity is creative depends on perspectives of the society or the public. These features suggest that creativity is less likely to occur in societies with strong religiosity (Glăveanu, 2010).

Correlation analyses also showed that the overall religiosity was negatively related to RPI, and RPI was positively related to GCI. These results echo Cinnirella and Streb (2017) argument that religious tolerance has a positive relation with innovation and creativity. In addition, previous research finds that religiosity has a negative relationship with diffusion rate of innovation (Azam et al., 2011) and total factor productivity (Herzer and Strulik, 2016), providing indirect evidence for the “hinder hypothesis.” Here the “hinder hypothesis” should be given more consideration. In this study, we found that the negative effect of religiosity on creativity became insignificant when IQ and RPI were controlled, and the religiosity–creativity relationship is significant only in affluent countries. This suggests whether the “hinder hypothesis” holds depends on other factors (e.g., economy).

### The Effects of Different Denominations on National Creativity

Correlation and regression analysis in this study illustrated that both proportion of Catholics and proportion of Protestants had a positively correlation with GCI, while only the correlation analysis showed that proportion of Muslims had a negative correlation with GCI. Proportions of adherents of Orthodox and Buddhism had no significant relation with GCI. Thus Hypothesis 2 was supported. These results can account for the controversies over the religion–creativity association. That is, the effects of different denominational cultures on national creativity were dissimilar.

Religions can build social networks based on different religious traditions, doctrines, and values (Dana, 2009). This suggests that religions can influence creativity and innovation through norms, customs, and beliefs that are to some extend pervading (Herbig and Dunphy, 1998). Berry (1999) argued that different religious traditions had dissimilar value systems encouraging the adherents to attain achievements in different domains.

This study showed that two denominational cultures, namely Protestant and Catholic, had positive effects on creativity, supporting Hypothesis 2a and 2b. The positive effect of Protestant culture on creativity can be partly attributed to the Protestant work ethic. Weber (1930) pointed that Protestant work ethic that emphases hard work, discipline, and frugality was conducive to rapid development of economics and science. Individuals are religiously compelled to work hard to thrive in a secular career, facilitating the accumulation of capital. Berry (1999) further proposed that the emphasis laid on utilitarianism and disinterested inquiry into “Nature” were responsible for Protestant fruitfulness in science-related areas. Protestant culture also values individual choice, personal freedom, and self-actualization (Inglehart and Oyserman, 2004), which are contributive to innovation and creativity. Existing research finds that intrinsic motivations are usually associated with increased creativity, while extrinsic motivations are usually associated with decreased creative performance (Hennessey, 2003). Professional development, achievement, and wealth accumulation advocated by Protestant work ethic can stimulate more intrinsic motivations in Protestants to achieve maximized personal value. Westwood and Low (2003) argued that achievement orientation and individualism in Protestant culture are beneficial for creativity and innovation. Consistently, this positive connection between individualism and creativity or innovation has been supported by much national level research (e.g., Shane, 1993; Efrat, 2014).

Recently, some researchers hold that the work ethics such as hard working and thrifty have spread in Catholic world before Protestant Reformation (Parboteeah et al., 2009; Andersen et al., 2017). These ethics, which have translated into economic success and productivity growth, can partly account for the positive relationship between Catholic and creativity. Herbig and Dunphy (1998) proposed that the values conductive to creativity and innovation, such as achievement-orientation, materialism, and individualism, are not exclusive to Protestant. These propositions are supported empirically by Berry (1999) who found that Catholic may be as creative as Protestants.

Contrary to Protestant and Catholic, Islam has a negative relationship with national creativity, supporting Hypothesis 2c. This suggests that countries with a greater proportion of Islam adherents tend to have a lower level of creativity. In Koran, supreme power regulates everything, the duty of adherents is only to obey and follow faith and rules. The faith that Allah determined all and creates the entire world is deeply rooted in Islam culture, leading the believers to accept all givens and refuse to spontaneously seek to alter things (Westwood and Low, 2003). These traditions, in some sense, are disruptive to innovation and creativity. Herbig and Dunphy (1998) proposed that fatalism, non-secularism, and belief in absolute truth in the Islamic world impeded science and innovation. However, there are also studies showing that Islamic tradition has a positive impact on creativity in art-related areas (Lubart, 2010). Furthermore, in our regression model the negative effect of Islam on GCI was partialed out by IQ and RPI, indicating that the association between Islam and creativity need further investigation.

In line with the Hypothesis 2d, Buddhism had no relation with creativity. Asceticism advocated by Buddhism devalues materialism and productivity growth (Westwood and Low, 2003). This may counteract the positive effects of Buddhist practice such as mindfulness (Berkovich-Ohana et al., 2017) and meditation (Colzato et al., 2012; Ding et al., 2014) on creativity. However, the effects of Buddhism on creativity need deep investigations in future studies. Because Buddhism encourages the impartial investigation of nature, which is consistent with modern Western scientific and philosophic thought (Yong, 2005).

### Effect of GDPpc on the Relationship Between Religion and Creativity

This study found that GDPpc had a moderation effect on the religion–creativity relationship. Specifically, the overall religiosity–creativity association, the Protestant–creativity association, and the Islam–creativity association were all moderated by GDPpc. Further analysis showed that only in countries with high GDPpc the national creativity can be predicted by overall religiosity or denominational cultures (Protestant and Islam). Thus Hypothesis 3 was also supported.

It is surprising and interesting that no matter whether the religion–creativity relationship is positive or negative, the originally significant relationship lose its significance in low GDPpc countries. This can be account for by the fact that a considerable amount of patents belong to foreigners in low GDP countries (Raghupathi and Raghupathi, 2017). Fagerberg et al. (2010) also indicated that technologic advancement in developing countries mostly relies on “spillovers” of that of developed countries. In other words, the GCI scores of low GDP countries are not indicative of the creativity of the residents of these countries. A heavy reliance on the innovation and technology in foreign countries may have weakened the influences of religions/denominations on national creativity in low GDP countries.

The economies of less affluent countries are mostly in factor-driven or investment-driven stage, with innovation/creativity being less important in economic development, while the almost all of developed countries are in innovation-driven stage (Ozawa, 1992; Acs et al., 2008). In less affluent countries, foreign direct investment is an important impetus to GDP growth (Seyoum et al., 2015). Ozawa (1992) proposed that the foreign direct investment provided not only finance but also technology for developing countries. That is, domestic creativity of a developing country is largely invisible and occupies a small portion. But for affluent countries, creativity/innovation became the core impetus to economic growth, causing the fact that the relationship between creativity and its restraining or promoting factor starts to emerge, and that creativity/innovation mostly relies on domestic resources. These may partly account for the moderation effects of GDP in this study.

### Limitations and Future Directions

To our knowledge, there was only one research exploring the religion–creativity relationship at national level (Bénabou et al., 2013). We have made a significant progress in using more comprehensive indicators of religiosity and national creativity. In addition, we investigated the effects of different denominations on national creativity, as well as the moderation effect of economy in a larger sample of countries. But there are still some limitations.

First, although GCI used in this study is a more comprehensive indicator of creativity than that used in previous research, it still lacks creativity measures of other domains, such as music, literature, and painting. Various cultures encourage creativity in different areas (Westwood and Low, 2003). Findings in this study thus cannot be generalized to creativity in other areas. Future researchers are expected to explore whether religions/denominations are associated with creativity in these domains.

Second, this study has only explored the effects of limited numbers of denominations/religions in creativity. There are many denominations that are not included, such as Judaism. Judaism may be more strongly contributive to innovations in both science-related and art-related areas than other religious traditions (Berry, 1999). However, in the WVS dataset that comprises 340,297 responders, there are only 2,172 Judaists. Judaists contribute to a proportion that is too small to be used as an indicator of Judaism religious culture that may exert influence of national creativity. Maybe individual level study is more appropriate for investigating the Judaism–creativity association.

Third, this study was conducted at national level, with no individual level data to validate the research findings. We hope that this limitation can be overcome by future research. In the investigation of the effects of religion on social outcomes, individual level findings may collide with national level findings (Myers, 2012).

## Conclusion

The present study found that the overall religiosity has a negative relationship with national creativity, which is consistent with previous research. However, different denominations show dissimilar effects on creativity. Protestant and Catholic are positively related with national creativity, while Islam is negatively related with national creativity. This study also finds that the religion–creativity relationship at national level was moderated by GDPpc. Specifically, the influences of religions/denominations on creativity only exist in affluent countries. These results provide explanations for why there are paradoxical findings on the roles of religions in influencing creativity.

## Author Contributions

ZL collected and analyzed the data under the supervision of QG. QG and ZL designed the study. QG, RW, and ZW contributed reagents, materials, and analysis tools. ZL, QG, PS, and RW contributed to the writing of the manuscript. ZL, QG, and ZW contributed to the revision.

## Conflict of Interest Statement

The authors declare that the research was conducted in the absence of any commercial or financial relationships that could be construed as a potential conflict of interest.

## Footnotes

1 www.worldvaluessurvey.org

2 http://data.worldbank.org
